# Recurrent severe thrombocytopenia induced by anti-HER2 therapy in a breast cancer patient with an underlying immune disorder: a case report and literature review

**DOI:** 10.3389/fonc.2026.1795252

**Published:** 2026-03-10

**Authors:** Bo Wang, Lin Chen, Qian Tang

**Affiliations:** Department of Thyroid and Breast Surgery, Liuyang People’s Hospital, Liuyang, Hunan, China

**Keywords:** anti-HER2 therapy, autoimmune antibodies, breast cancer, case report, thrombocytopenia

## Abstract

This case report describes a 51-year-old female with HER2-positive breast cancer who developed recurrent, severe thrombocytopenia during treatment with trastuzumab and pertuzumab. Through a retrospective analysis of her entire treatment course—encompassing neoadjuvant, adjuvant, and radiotherapy phases—we dynamically observed the temporal correlation between anti-HER2 therapy administration and acute drops in platelet count (nadir: 8×10^9^/L), accompanied by bleeding symptoms. The thrombocytopenia responded well to thrombopoietin-stimulating agents and immunomodulatory therapy but recurred persistently, even after switching to trastuzumab monotherapy or its subcutaneous formulation. Laboratory workup was notable for revealing a predisposition to undifferentiated connective tissue disease (UCTD) with positive antinuclear antibody (ANA) and positive anti-SSA/Ro52 antibodies. Ultimately, all targeted therapies were discontinued due to intolerability. This case highlights that both trastuzumab and pertuzumab (including subcutaneous forms) can induce rare immune-mediated thrombocytopenia, a risk significantly heightened by underlying autoimmune serology. The mechanisms appear multifactorial, involving the patient’s immune status, treatment phase, and route of administration. It underscores the need for heightened clinical vigilance, prompt drug suspension, supportive care, and individualized, multidisciplinary management in such scenarios.

## 1 Introduction

HER2-positive breast cancer accounts for approximately 15-20% of all breast cancer cases and is characterized by aggressive behavior and poorer prognosis (1). The advent of anti-HER2 monoclonal antibodies, such as trastuzumab and pertuzumab, has revolutionized the therapeutic landscape, significantly improving disease-free and overall survival for these patients (2). However, treatment-related adverse events remain a critical challenge in clinical management. Among these, thrombocytopenia is a notable concern.

Chemotherapy-induced thrombocytopenia (CIT) is relatively common in patients with solid tumors. Severe cases can lead to treatment delays, dose reductions, or even discontinuation, ultimately compromising disease control and survival outcomes (3, 4). The pathogenesis of CIT is multifactorial, involving bone marrow suppression, immune-mediated mechanisms, and direct drug toxicity. Conventional supportive measures, including thrombopoietin analogs, platelet transfusions, and novel TPO receptor agonists, are employed but may have limited efficacy or raise safety concerns (5). In contrast to chemotherapy, severe thrombocytopenia induced by HER2-targeted agents is exceedingly rare in breast cancer patients. It is poorly characterized in the literature, with an unclear mechanism. Notably, while registry data report an incidence of any-grade thrombocytopenia between 1.5% and 3% (6), the risk of severe, life-threatening episodes is often underestimated in clinical practice (7).

Furthermore, immune dysregulation is closely linked to tumorigenesis, progression, and treatment-related toxicities. Undifferentiated connective tissue disease (UCTD) refers to a chronic condition featuring autoimmune manifestations that do not meet the diagnostic criteria for a specific rheumatic disease (8). Patients with UCTD may experience immune-related complications or altered drug reactions during oncologic therapy, adding complexity to the management of hematological adverse events (9). The co-occurrence of breast cancer and subclinical immune dysregulation, while uncommon, poses diagnostic and therapeutic challenges. In such patients, the emergence of severe, unexplained thrombocytopenia during chemotherapy or targeted therapy warrants vigilance for multifactorial interactions, including underlying autoimmunity, drug hypersensitivity, and bone marrow injury (10).

This case is distinctive and instructive. It documents recurrent severe thrombocytopenia in a patient with HER2-positive breast cancer receiving dual anti-HER2 therapy (trastuzumab plus pertuzumab, including the subcutaneous formulation) throughout the entire treatment course—neoadjuvant, adjuvant, and radiotherapy phases. The patient had underlying immune dysregulation (positive ANA and anti-SSA/Ro52 antibodies), which may be a key predisposing factor. This report highlights the complex interplay between treatment phase, route of administration, and the patient’s immune background, offering a novel perspective on the mechanism and management of this adverse event.

## 2 Case report

A 51-year-old female presented in May 2025 with a two-month history of a right breast mass. She had no significant past medical history or family history of malignancy. Physical examination revealed a 3×3 cm firm mass at the 10–11 o’clock position of the right breast and palpable right axillary lymphadenopathy. Core needle biopsy confirmed invasive ductal carcinoma (histologic grade II). Immunohistochemistry showed ER positivity (40%), PR negativity, HER2 (3+), and Ki-67 (30%). The clinical stage was cT2N1M0 (stage IIB). Written informed consent was obtained from the patient for the publication of this case report and any accompanying data, which have been anonymized to protect her privacy.

### 2.1 Neoadjuvant phase

Prior to treatment, the patient’s baseline platelet count was 257×10^9^/L. On July 8, 2025, she commenced neoadjuvant therapy with the TCbHP regimen (nab-paclitaxel, carboplatin, trastuzumab, and pertuzumab). Two days post-treatment (July 10, 2025), a dramatic drop in platelet count to 16×10^9^/L was observed, which further declined to a nadir of 8×10^9^/L on day 5 post-treatment, accompanied by gingival bleeding and skin petechiae. She was managed aggressively with recombinant human thrombopoietin (rhTPO), eltrombopag, interleukin-11 (IL-11), and platelet transfusions, leading to recovery. This severe episode was initially attributed to chemotherapy. The patient declined further neoadjuvant chemotherapy, and the decision was made to proceed with targeted therapy alone.

### 2.2 Surgical intervention and perioperative event

Following the patient’s refusal of further chemotherapy, she received a second cycle of dual HER2-targeted therapy (trastuzumab plus pertuzumab) on July 29, 2025, and underwent a right modified radical mastectomy the next day. The postoperative pathology revealed a Miller & Payne grade 3 treatment response with only 10% residual tumor and one involved lymph node out of ten (1/10), indicating a good therapeutic response. At this point, targeted therapy was not considered a cause for thrombocytopenia. However, on postoperative day 7 (August 5, 2025), the patient developed bruising at the incision site and bloody drainage. A blood test showed a platelet count of 94×10^9^/L. The platelet count recovered to 158×10^9^/L within three days after oral administration of leucogen, and the local symptoms resolved.

### 2.3 Adjuvant phase and recurrent thrombocytopenia

Adjuvant dual HER2-targeted therapy was initiated postoperatively, but thrombocytopenia recurred repeatedly. Following the third cycle, the platelet count dropped to 81×10^9^/L on day 1 post-infusion and reached a nadir of 47×10^9^/L by day 3. It then gradually recovered after IL-11 treatment, normalizing within a week. For the fourth cycle, in an attempt to mitigate the risk — motivated by the hypothesis that altering the administration route might reduce thrombocytopenia risk alongside the patient’s preference — the subcutaneous formulation of pertuzumab/trastuzumab (Phesgo^®^) was administered. The platelet count decreased to 52×10^9^/L by day 3 post-treatment, falling further to its nadir of 32×10^9^/L on day 5. Recovery was achieved within a week with IL-11 and leucogen. Subsequently, the patient opted to return to the intravenous formulation.

### 2.4 Multidisciplinary consultation and autoimmune workup

Given the recurrent and unexplained thrombocytopenia, a multidisciplinary team (MDT) consultation was held. To exclude other etiologies, an autoimmune panel was performed. The results were notable for a positive antinuclear antibody and were positive for anti-SSA/Ro52 antibodies. A minor salivary gland biopsy showed chronic inflammation, insufficient for a diagnosis of Sjögren’s syndrome. This constellation—subjective symptoms (e.g., arthralgias) alongside serologic evidence of autoimmunity (positive ANA and positive anti-SSA/Ro52)—aligned with the clinical diagnosis of undifferentiated connective tissue disease (UCTD), as established by the consulting rheumatologists. Hydroxychloroquine was subsequently initiated.

### 2.5 Radiotherapy phase

During subsequent radiotherapy, given the history of severe thrombocytopenia, pertuzumab was withdrawn, and trastuzumab monotherapy was attempted. However, severe thrombocytopenia recurred with each administration. Following the fifth cycle on October 1, the platelet count dropped markedly and reached a nadir of 37×10^9^/L three days later. After the sixth cycle on October 22, a more precipitous decline occurred, with the platelet count falling to its lowest point of 10×10^9^/L within two days. Each episode required intervention with rhTPO, IL-11, and glucocorticoids for recovery. The patient completed the full course of radiotherapy during this period.

### 2.6 Subsequent treatment and final attempt

After radiotherapy, a seventh cycle of trastuzumab was planned. On November 13, 2025, the pre-treatment platelet count was 137×10^9^/L. Prophylactic eltrombopag was administered to elevate the baseline count, which increased to 149×10^9^/L by November 18, 2025. Trastuzumab was administered on that day. Alarmingly, the platelet count plummeted to 19×10^9^/L on the following day (November 19). Recovery was achieved with rhTPO, glucocorticoids, and leucogen over ten days.

### 2.7 Treatment modification and outcome

**Figure 1:**
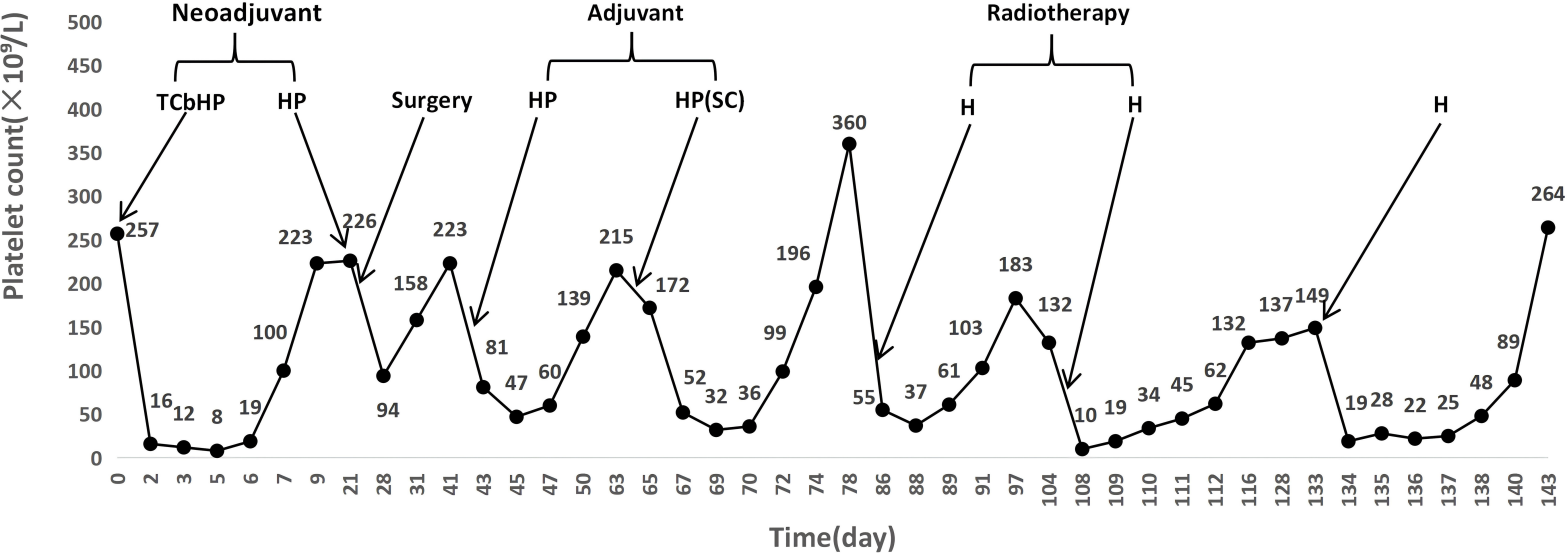
Temporal profile of platelet counts and anti-HER2 therapy administration. Abbreviations: TCbHP, nab-paclitaxel, carboplatin, trastuzumab, and pertuzumab; HP, trastuzumab plus pertuzumab (intravenous); Surgery, right modified radical mastectomy; HP(SC), fixed-dose subcutaneous combination of pertuzumab and trastuzumab (Phesgo^®^); H, trastuzumab monotherapy. The graph illustrates dynamic platelet count changes, with arrows marking key interventions.

Despite the use of prophylactic and therapeutic agents, each episode of thrombocytopenia became more severe and the recovery period progressively lengthened, indicating an unacceptable risk of hemorrhage. The patient declined any further HER2-targeted therapy. Upon permanent discontinuation of all anti-HER2 agents, the platelet count stabilized within the normal range. The patient subsequently continued on letrozole for endocrine therapy alone, with no further hematologic abnormalities during follow-up. The detailed treatment timeline and corresponding platelet dynamics are summarized in **Table 1** and visually presented in **Figure 1**. Notably, the patient’s severe thrombocytopenia occurred in isolation, with white blood cell counts and hemoglobin levels remaining consistently normal throughout all treatment phases. This pattern argues against generalized bone marrow suppression and supports a drug-specific, suspected immune-mediated mechanism.

**Table 1:** Summary of treatment timeline and platelet dynamics.

Treatment phase	Date (2025)	Intervention	Platelet nadir (×10^9^/L) & timing	Management	Outcome
Neoadjuvant	Jul 8	TCbHP (Cycle 1)	8 (Day 5 post-TCbHP)	rhTPO, Eltrombopag, IL-11, Transfusion	Recovery; chemo declined
Surgery	Jul 29	HP (Cycle 2) → Surgery	94 (Day 7 post-HP)	Oral Leucogen	Rapid recovery
Adjuvant	Aug 19	HP (Cycle 3)	47 (Day 3 post-HP)	IL-11	Recovery in 1 week
Adjuvant	Sept 10	Phesgo^®^ SC (Cycle 4)	32 (Day 5 post-Phesgo)	IL-11, Leucogen	Recovery in 1 week
Radiotherapy	Oct 1	Trastuzumab (Cycles 5)	37 (Day 3 post-H)	rhTPO, IL-11, Glucocorticoids	Recovery; RT completed
Radiotherapy	Oct 22	Trastuzumab (Cycles 6)	10 (Day 2 post-H)	rhTPO, IL-11, Glucocorticoids	Recovery; RT completed
Subsequent Treatment	Nov 18	Trastuzumab + Prophylaxis (Cycle 7)	19 (Day 1 post-H)	rhTPO, Glucocorticoids, Leucogen	Recovery in 10 days
Final	Post Nov 2025	All targeted therapy discontinued	Remained stable	Letrozole only	No further events

## 3 Discussion

### 3.1 Literature context and comparative analysis of case features

This case exhibits both typical and unique characteristics of anti-HER2 therapy-associated thrombocytopenia. To contextualize our findings within the broader literature, we have summarized key recent reports, including the present case, in **Table 2** (7, 11–15). A comparative analysis reveals several important patterns and insights.

**Table 2:** Comparative analysis of reported cases of severe thrombocytopenia associated with anti-HER2 targeted therapy.

Reporter (Year)	Patient age	Triggering agent	Platelet nadir (×10^9^/L)	Time to onset (Post-dose)	Primary management	Immune status	Notable findings/remarks
Present Case (2025)	51	Trastuzumab + Pertuzumab	8-10	1–3 days, recurrent episodes	TPO, IL-11, Corticosteroids, Eltrombopag, Transfusion	UCTD	Positive autoimmune antibodies; Includes subcutaneous formulation
Huff et al. (2022) (7)	36	Trastuzumab + Pertuzumab	5	7 days after 1st dose	Transfusion, then trastuzumab stopped	Not reported	Successfully completed therapy with pertuzumab monotherapy
Wang et al. (2021) (11)	52	Trastuzumab	1	1 day after 1st dose	Corticosteroids, Transfusion	Not reported	Failed rechallenge with a reduced weekly schedule
Miarons et al. (2016) (12)	70	Trastuzumab	39	Gradual decline after 4th cycle	Corticosteroids	Not reported	Presented as a slow, progressive decline
Pino et al. (2013) (13)	70	Trastuzumab	0	<24 hours upon re-dosing	Corticosteroids, IVIG, Transfusion	Not reported	Occurred during re-treatment for recurrence 4 years after adjuvant therapy
Mantzourani et al. (2011) (14)	56	Trastuzumab	5	3 days after 1st dose	IVIG	Not reported	No recurrence after rechallenge; completed 1-year therapy
Zeng et al. (2020) (15)	29	Trastuzumab	3	6 hours post-dose	TPO, IL-11, Transfusion, Corticosteroids	Not reported	Rapid onset; complicated by uterine bleeding

#### 3.1.1 Triggering agent

Early reports primarily implicated trastuzumab monotherapy (11–15). However, the report by Huff et al. (7) clearly identified pertuzumab as a potential trigger, suggesting that this may be a class effect of anti-HER2 monoclonal antibodies rather than a trastuzumab-specific phenomenon. Our case further supports this, as severe thrombocytopenia occurred with both the dual-target regimen and with attempts at monotherapy.

#### 3.1.2 Clinical presentation and timing

The overwhelming majority of cases, including ours, present with acute, severe thrombocytopenia (nadir often <20×10^9^/L) occurring within 24 hours to one-week post-infusion. This rapid onset strongly aligns with a suspected immune-mediated mechanism. The recurrent nature of thrombocytopenia in our patient, tightly correlated with each administration, reinforces the drug’s role as the precipitating factor. As clearly illustrated in **Figure 1**, each sharp decline in platelet count was temporally associated with an administration of anti-HER2 therapy. Despite this clear temporal association, the decision to proceed with repeated rechallenges was guided by the imperative to maintain this curative-intent, dual HER2-blockade regimen for high-risk breast cancer, as alternative strategies considered by the multidisciplinary team (e.g., T-DM1) were deemed unsuitable for the adjuvant setting. This risk-benefit calculation, made in close consultation with the patient who strongly desired to continue potentially curative therapy, represented a shared decision.

#### 3.1.3 Management and outcome

Management and Outcome: Most cases respond well to interventions such as corticosteroids and/or intravenous immunoglobulin (IVIG), yet thrombocytopenia typically recurs upon rechallenge, leading to permanent therapy discontinuation (11–13). This pattern was echoed in our patient’s course. Our pragmatic management, including the use of glucocorticoids in later cycles which reflected the evolving hypothesis of an immune-mediated mechanism, was guided by principles for secondary immune thrombocytopenia. Notably, IVIG was not employed, and the failure of prophylactic eltrombopag in Cycle 7 underscores a potent, acute, drug-dependent immune destruction distinct from typical chemotherapy-induced thrombocytopenia, thereby reinforcing the immune-mediated nature of this toxicity.

### 3.2 Distinctive findings and novel insights from the present case

Unique Autoimmune Background and Mechanistic Implications: The most salient feature of our case is the patient’s positive ANA and positive anti-SSA/Ro52 antibodies status and clinical predisposition to undifferentiated connective tissue disease (UCTD). To our knowledge, none of the previously reported cases have specifically highlighted or confirmed a pre-existing autoimmune serological profile. The attribution to anti-HER2 therapy is based on thrombocytopenia recurring exclusively upon re-exposure to these agents after chemotherapy cessation, a temporal pattern distinct from classic myelosuppression and suggestive of an suspected immune-mediated mechanism. This finding directs attention to a deeper pathophysiological hypothesis: a pre-existing immune dysregulation may significantly increase a patient’s susceptibility to suspected immune-mediated hematologic toxicity from targeted agents (16). In autoimmune conditions, underlying immune dysregulation can create a predisposing inflammatory milieu. The introduction of a biologic agent like an anti-HER2 antibody may then act as a “second hit,” potentially triggering a drug-dependent, ITP-like immune response against platelets. This mechanistic framework aligns with the patient’s sustained remission after therapy cessation. It provides a crucial clue for understanding the susceptibility mechanisms behind this rare adverse event and raises the hypothesis that screening for relevant autoantibodies in patients with unexplained thrombocytopenia or subtle autoimmune features might help identify those at higher risk, prompting consideration of closer monitoring. Accordingly, for patients with a known or newly identified autoimmune serological profile, drug-related immune mechanisms should be considered high in the differential diagnosis of treatment-emergent cytopenias.

Dynamic Observation Across the Entire Treatment Continuum and the Novel Context of “Radiotherapy with Targeted Therapy”: Unlike most literature describing isolated events in a single treatment phase (e.g., neoadjuvant or adjuvant), this report provides a systematic, dynamic account of recurrent thrombocytopenia throughout the entire therapeutic journey—from neoadjuvant and adjuvant settings into the radiotherapy phase. A particularly unique observation is the occurrence of severe thrombocytopenia during radiotherapy while the patient was receiving targeted therapy. This finding addresses a gap in the literature, indicating that the hematologic toxicity risk from targeted agents remains high even during radiotherapy, a phase with potential superimposed myelosuppressive effects (17). It provides evidence to guide vigilant monitoring and management during this specific period.

This case extends the existing safety profile of anti-HER2 therapy. Specifically, our patient experienced severe thrombocytopenia following administration of the fixed-dose subcutaneous formulation (Phesgo^®^). As all previously reported cases are associated with intravenous administration (18), this provides clinical evidence that the subcutaneous route can independently induce this serious reaction, with important implications for the comprehensive safety evaluation of this formulation.

### 3.3 Impact on clinical decision-making and management strategy

Recurrent thrombocytopenia necessitated a shift from standard care to active, integrated toxicity management. This encompassed intensive monitoring, preemptive thrombopoietic/immunomodulatory support, and empirical formulation trials—all driven by the imperative to preserve curative-intent dual HER2-targeted therapy, thereby minimizing recurrence risk, through collaborative, patient-centered decision-making that balanced treatment benefit against toxicity. This experience demonstrates that with vigilant monitoring and proactive support, life-saving therapy can often be sustained despite severe toxicity, highlighting a dynamic management model for navigating the trade-off between efficacy and risk in complex scenarios.

### 3.4 Limitation

In reviewing this case, its limitations must be acknowledged. Firstly, the patient declined bone marrow biopsy, which was based on patient preference and the rapid clinical response to corticosteroids, an indirect indicator of an immune-mediated etiology, so the precise pathological mechanism of thrombocytopenia could not be definitively confirmed. Furthermore, as a single case report, its generalizability is inherently limited. Specialized diagnostic tests, such as assays for drug-dependent anti-platelet antibodies, measurement of the immature platelet fraction, and the post-transfusion platelet count at 1 hour, were not available or performed, which represents a further limitation in characterizing the precise mechanism of thrombocytopenia. The diagnosis and follow-up criteria for UCTD also lack standardization, underscoring the need for larger cohort studies and evidence-based guidelines (19, 20). Future research should focus on multicenter studies and systematic follow-up to elucidate the immune mechanisms of precision therapy-related thrombocytopenia in breast cancer and the prognosis of UCTD in this context, thereby refining personalized intervention strategies.

## 4 Conclusion

This case constructs a more complex clinical picture by elucidating three underreported features: the autoimmune background, the association of thrombocytopenia across the entire treatment continuum (neoadjuvant, adjuvant, and radiotherapy), and the risk confirmed with the subcutaneous formulation. It underscores the pivotal role of a multidisciplinary team (MDT) in managing such complexity, where pathogenesis involving the tumor, therapeutic agents, and underlying immune dysregulation necessitates collaborative strategies—including thrombopoietin receptor agonists, IL-11, corticosteroids, and immunomodulatory therapy (e.g., hydroxychloroquine) (21). Dynamic adjustments, challenged by patient compliance and bleeding risk, highlight the need for ongoing MDT communication and a structured follow-up protocol involving breast surgery, rheumatology, and oncology/hematology to monitor parameters and individualize management. Ultimately, this report highlights the critical importance of MDT collaboration, individualized dynamic management, and long-term follow-up in the precision therapy era, providing a valuable reference for managing similar complex scenarios involving immune dysregulation and severe hematologic adverse events.

## Data Availability

The original contributions presented in the study are included in the article/Supplementary Material. Further inquiries can be directed to the corresponding author.
